# Meta-assembly of genomic associations to identify cattle fat depot candidate genes and pleiotropic effects

**DOI:** 10.1186/s12864-024-11159-4

**Published:** 2024-12-24

**Authors:** Junpeng Yao, Cynthia D.K. Bottema, Mehar Singh Khatkar

**Affiliations:** School of Animal and Veterinary Sciences, The University of Adelaide, Roseworthy Campus, Roseworthy, South Australia 5371 Australia

**Keywords:** Meta-analysis, Quantitative trait loci, Bovine, Adipose, GWAS, SNP, Pleiotropy

## Abstract

**Background:**

Fat traits in cattle are considered important due to their contribution to beef eating quality and carcass economic value. Discovering the genes controlling fat traits in cattle will enable better selection of these traits, but identifying these genes in individual experiments has proven difficult. Compared to individual experiments, meta-analyses allow greater statistical power for detecting quantitative trait loci and identifying genes that influence single and multiple economically important fat traits.

**Results:**

This meta-analysis study focussed on fat traits related to the major adipose depots in cattle (namely, carcass fat, intramuscular fat, internal fat, intermuscular fat, and subcutaneous fat) and was conducted using data from the Animal Quantitative Trait Loci (QTL) database. There were more Meta-QTL regions for intramuscular fat and subcutaneous fat (*n* = 158 and *n* = 55 regions, respectively) and far fewer for carcass fat and internal fat (*n* = 2 regions each). There were no Meta-QTL regions found for intermuscular fat. Of these 216 Meta-QTL regions, only 16 regions overlapped and affected two or more fat depots. The number of genes found for the fat depots was reflected in the size and number of the Meta-QTL regions (*n* = 20, 84, 1336 and 3853 genes for the carcass, internal, subcutaneous and intramuscular fat, respectively). The identification of these QTL allowed a more refined search for candidate genes. For example, the 232 genes in the Meta-QTL regions for carcass fat on BTA2, for intramuscular fat on BTA12, and the overlapping Meta-QTL regions on BTA2, BTA5, and BTA6 were readily screened, and 26 candidate genes were nominated based on their physiological roles using the GeneCards and DAVID databases.

**Conclusions:**

The number of Meta-QTL regions for the various fat depots was relative to the number of associations in the database. However, the scarcity of overlapping Meta-QTL regions suggests that pleiotropic gene variants, which control multiple fat depots in cattle, are rare. The identification of candidate genes in the Meta-QTL regions will improve our knowledge of the genes with regulatory functions in adipose metabolism affecting meat quality and carcass economic value.

**Supplementary Information:**

The online version contains supplementary material available at 10.1186/s12864-024-11159-4.

## Background

Adipose tissues play important roles in energy storage and metabolism and physical protection, as well as biological regulation by producing endocrine and paracrine hormones in animals, including cattle [1, 2]. In cattle, adipose tissues are classified into two groups based on their location: physically separable fat and physically inseparable fat. The separable fat depots include subcutaneous fat, intermuscular fat and internal fat, and their size can be used as visual measures to predict the proportion of carcass fat to meat to determine carcass quality [3]. The remaining fat depot is intramuscular fat (IMF), also called marbling fat, which is highly valued in the USDA (the United States Department of Agriculture’s Agricultural Marketing Service) and MSA (Meat Standards Australia) beef grading systems because the level of IMF is related to meat eating quality [4–7]. As cattle breeds differ greatly in amount of fat in these various depots, the genetics of fat traits in cattle has been explored and genes with causative variants that control fat traits have been targeted in quantitative trait loci (QTL) and single nucleotide polymorphism (SNP) association studies [8]. However, pleiotropic effects of these QTL and candidate genes on cattle fat depots has not been described and genes which may be controlling more than one fat depot need to be explored further.

Meta-analysis is emerging as an important tool for the quantitative aggregation and synthesis of knowledge from independent studies on the same or a similar subject. Pooling of results from several studies allows greater statistical power for QTL detection and more precise estimation of their genetic effects. Hence, by comparing the results from many genetic linkage mapping experiments and genome-wide association studies, a meta-analysis can yield conclusions that are stronger than those of individual studies and can give greater insight into the genetic architecture of complex traits. Recently, meta-analyses have been conducted for the validation of QTL regions for production, reproduction and adaptation traits in cattle [9, 10]. However, the approach has not yet been used for the fat traits in cattle. Herein, a meta-analysis was conducted to identify the consensus QTL regions and strength of the associations across the bovine genome with a particular focus on fat-related traits.

The Animal Quantitative Trait Loci (QTL) database (Animal QTLdb) (https://www.animalgenome.org/cgi-bin/QTLdb/index) provides comprehensive and up-to-date information reported in the scientific literature on QTL and SNPs associated with livestock phenotypes [11]. Using the web-viewer and other tools in Animal QTLdb, researchers can locate QTL and SNPs obtained from the genetic linkage analyses and genome-wide association scans (GWAS) and then manually compare QTL segments across studies in order to identify candidate genes potentially controlling the phenotype of interest [12].

However, the whole process to mine the QTL and SNP data in such unprecedented volume requires careful analysis for gene discovery. Many results in the Animal QTLdb are also not readily comparable as the models used in statistical analysis have different assumptions and inferences. Thus, to improve the discovery of causal DNA variants, a more efficient and reliable statistical method, known as QTL meta-analysis, has been implemented [12–15]. In the genomic research on *Drosophila*, there have been two simple and practical meta-analysis methods to visualize the most likely QTL region in a chromosome [16]. The first analytical method is to create a QTL counts plot at every centiMorgan (cM) of each chromosome, while the other method is to use the kernel density distribution plot of the counts. However, if the goal is to combine QTL and SNP associations across studies, then the method must take the significance of the QTL and SNPs into account. This approach was used herein to identify Meta-QTL regions for fat deposition in cattle. The goal of this meta-assembly was to determine if the same or different genes control the adipogenesis of different fat depots in cattle. Therefore, the fat traits with phenotypic records used in the meta-assembly included measures of intramuscular fat, intermuscular fat, subcutaneous fat, internal fat and carcass fat. The identified Meta-QTL regions were also utilised to better identify positional candidate genes.

## Methods

The association data with 19,720 QTL and SNPs from 236 publications were retrieved from Cattle Quantitative Trait Locus (QTL) Database (Cattle QTLdb) (https://www.animalgenome.org/cgi-bin/QTLdb/BT/index, accessed August 2023). All QTL and SNP coordinates were based on the ARS-UCD1.2 bovine reference genome assembly. To maintain consistency in the analysis, gene annotations were performed using the same reference assembly. The fat traits with phenotypes included measures of intramuscular fat, intermuscular fat, subcutaneous fat, internal fat and carcass fat. The meta-analysis strategies were adopted from Khatkar et al. [10]. To facilitate the analysis, the downloaded data were analysed in R studio 1.4.1717 [17]. A file with a subset of the data that exclusively included traits related to fat was created. For the individual traits, the corresponding number of associations recorded for each trait was counted (Table 1). In addition, these traits were grouped into broad categories, based on the fat depot location, using a configuration file (Table 1), which was utilized for subsequent analysis and plotting.

**Table 1:** Fat traits with the number of reported associations (QTL and SNPs) and assigned trait groups

QTLdb Trait ID	Trait name	Number of publications	Number of associations	Trait group
1228	Fat percentage	9	18	Carcass fat
1108	Fat trim yield	1	2	Carcass fat
1328	Fat weight	5	11	Carcass fat
1721	Fat area to ribeye area ratio	1	2	Intramuscular fat
1103	Intramuscular fat	24	140	Intramuscular fat
1027	Marbling score	70	2042	Intramuscular fat
1207	Meat fat content	2	2	Intramuscular fat
1333	Intermuscular fat percentage	2	6	Intermuscular fat
1330	Channel fat weight	1	1	Internal fat
1318	Internal fat weight	2	5	Internal fat
1350	Kidney fat weight	2	7	Internal fat
1089	Kidney, pelvic, heart fat percentage	11	67	Internal fat
1346	Kidney, pelvic, heart fat weight	1	4	Internal fat
1357	Pericardial fat weight	1	1	Internal fat
1334	Fat cover	6	35	Subcutaneous fat
1275	Fat thickness at the 12th rib	37	264	Subcutaneous fat
1109	Rib fat	2	3	Subcutaneous fat
1300	Sirloin fat depth	2	2	Subcutaneous fat
7322	Subcutaneous fat percentage	2	3	Subcutaneous fat
1029	Subcutaneous fat thickness	48	580	Subcutaneous fat

**Fig. 1:**
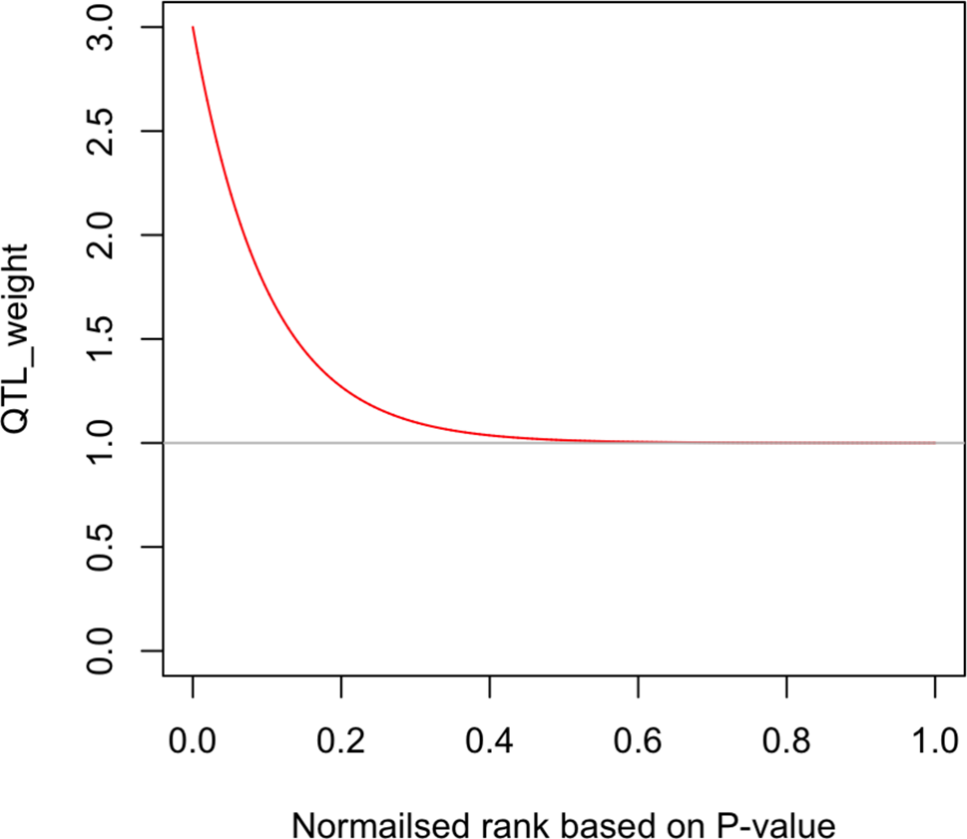
The weights assigned to the associations based on P-values

The reported significance levels (P-values) of QTL and SNPs were transformed into weights for the purposes of prioritizing the most significant associations in subsequent meta-analysis steps. The weights were calculated as follows: firstly, the QTL or SNP based on their reported P-values were ordered to determine their rank within the fat QTL dataset, and then, the ranks were normalized into a fixed range of 0 to 1 using min-max scaling normalisation. Finally, the weight of each QTL (QTL_weight) was computed using an exponential function with a constant decay rate across the normalized ranks. The equation for calculating the QTL weight was QTL_weight = 2 * e^ (-b * normalised_rank) + 1. In this equation, QTL_weight is the weight of the quantitative trait locus (QTL), e represents the base of the natural logarithm (approximately 2.718), b is the constant decay rate where b = 10, and normalised_rank is the rank of QTL based on the P-value. Based on the equation, the QTL weights are 3 for the QTL with the lowest P-values (most significant) and decline to 1 for the least significant (Fig. 1). This exponential weighting scheme ensures systematic prioritisation of the strongest statistical support by assigning greater weights to the smaller proportion and most significant SNPs, while assigning lesser weight to SNPs with a lower level of significance, thereby optimising the balance between significance and inclusivity in the meta-analysis.

For each trait or group of traits, the QTL and SNP association results within a one mega base (Mb) window with a sliding window size of 500 kilobases (Kb) were combined [18]. The QTL_weight of all the QTL and SNP associations within each window was summed and referred to as a “Meta-QTL score”. To identify prominent regions and visualize them in genome-wide plots, the Meta-QTL score was recorded against the central position of each window. The Manhattan and chromosome-wise plots were generated to display the Meta-QTL scores against the corresponding map positions on each chromosome. The prominent QTL positions in Manhattan and chromosome-wise plots with thresholds ≥ 3 were obtained and identified by examining the Meta-QTL map data. Overlapping Meta-QTL for all fat traits were extracted manually by comparing the start position and end position of each QTL for adjoining regions.

To annotate the Meta-QTL, the Ensembl database with ARS-UCD 1.2 reference genome assembly was used (https://asia.ensembl.org/index.html, accessed July 2023) in R studio 1.4.1717 with biomaRt package [17]. Ensembl gene IDs for each Meta-QTL position were identified using biomaRt, including 500 Kb flanking sequences on both sides. For each gene, the basic information was extracted, including the gene name, chromosome number, start and end positions (in megabases), and gene description.

As examples of the identification of positional candidate genes, for the top ranked Meta-QTL for carcass fat on BTA2 and for IMF on BTA12, the genes in the 1 Mb window of the peak were identified using the Ensembl database. Then, these gene lists were screened for potential candidate genes based on their biological functions using the Gene Cards database with the key words: “adip” for adipose, adipocyte, adipogenesis, “lip” for lipid metabolism, lipogenesis, liposarcoma, lipoma, “myo” for myogenesis, myocyte, myoblast, and “mus” for muscular, muscle (https://www.genecards.org/, accessed January 2024). Functions of the potential candidate genes were verified using DAVID (https://david.ncifcrf.gov/, accessed February 2024). Analogous to the above examples, candidate genes were also identified using the same process for the Meta-QTL that overlapped between the fat trait groups.

## Results

### Meta-QTL

Using the data from cattle Animal QTL database, a meta-analysis was conducted for bovine chromosomal regions associated with five cattle fat depots. The trait data were grouped for the relevant depot, and included carcass fat, intramuscular fat, intermuscular fat, internal fat and subcutaneous fat (Table 1). For each trait group, the QTL and SNP association results within a one mega base (Mb) sliding window were combined. The associations were weighted based on the number and significance of the associations at each position to derive a QTL score. The QTL were ranked based on their scores, and the QTL with thresholds ≥ 3 were considered to be highly significant “Meta-QTL” and were noted for each trait group. Both Meta-QTL in the 1 Mb windows and regions with adjacent Meta-QTL windows were identified (Tables 2 and 3, Supplementary Table S1). The overlapping Meta-QTL, which were significantly associated with more than one fat trait group, were also noted.

**Table 2:** Summary of Meta-QTL of top two ranked QTL listed for each fat trait group

Fat traits	# reported associations	# Meta-QTL	Chromosome (BTA)	Position (Mb)	QTL score
Carcass fat	31	2	2	3.5	3.01
			2	6.5	3.01
Intramuscular fat	2186	315	12	18.0	7.10
			12	18.5	164.73
			12	19.0	223.31
			12	19.5	87.88
			12	20.0	57.53
			12	20.5	34.32
			12	23.0	10.00
			12	23.5	8.01
			12	29.5	9.29
			12	30.0	16.35
			12	30.5	8.48
			17	23.5	3.00
			17	24.0	5.20
			17	24.5	5.40
			17	25.0	8.90
			17	25.5	5.70
			17	37.5	3.40
			17	38.0	3.40
			17	60.5	63.40
			17	61.0	65.70
			17	70.0	3.20
			17	70.5	5.00
Internal fat	85	4	1	31.0	3.01
			1	31.5	3.01
			5	56.0	3.00
			5	56.5	3.00
Intermuscular fat	6	0	0	0	0
Subcutaneous fat	887	107	6	1.0	3.00
			6	1.5	3.01
			6	36.5	3.01
			6	37.0	60.49
			6	37.5	168.22
			6	38.0	120.74
			6	38.5	11.00
			13	48.0	7.00
			13	48.5	6.00
			13	61.5	16.00
			13	62.0	31.01
			13	62.5	16.00

**Table 3:** Number of genes in Meta-QTL regions for the different fat trait groups

Fat trait group	# Publications	# Meta-QTL^a^	# Meta-QTL regions^b^	# Genes in Meta-QTL^c^
Carcass fat	15	2	2	20
Intramuscular fat	97	315	158	3853
Internal fat	2	4	2	84
Intermuscular fat	18	0	0	0
Subcutaneous fat	97	107	55	1336
Overlapping QTL	-	39	16	311

^a^ # Meta-QTL based on 1 Mb window

^b^ # Meta-QTL regions = # directly adjacent Meta-QTL windows + # individual Meta-QTL windows

^c^ # Genes in Meta-QTL = known & predicted genes

**Fig. 2:**
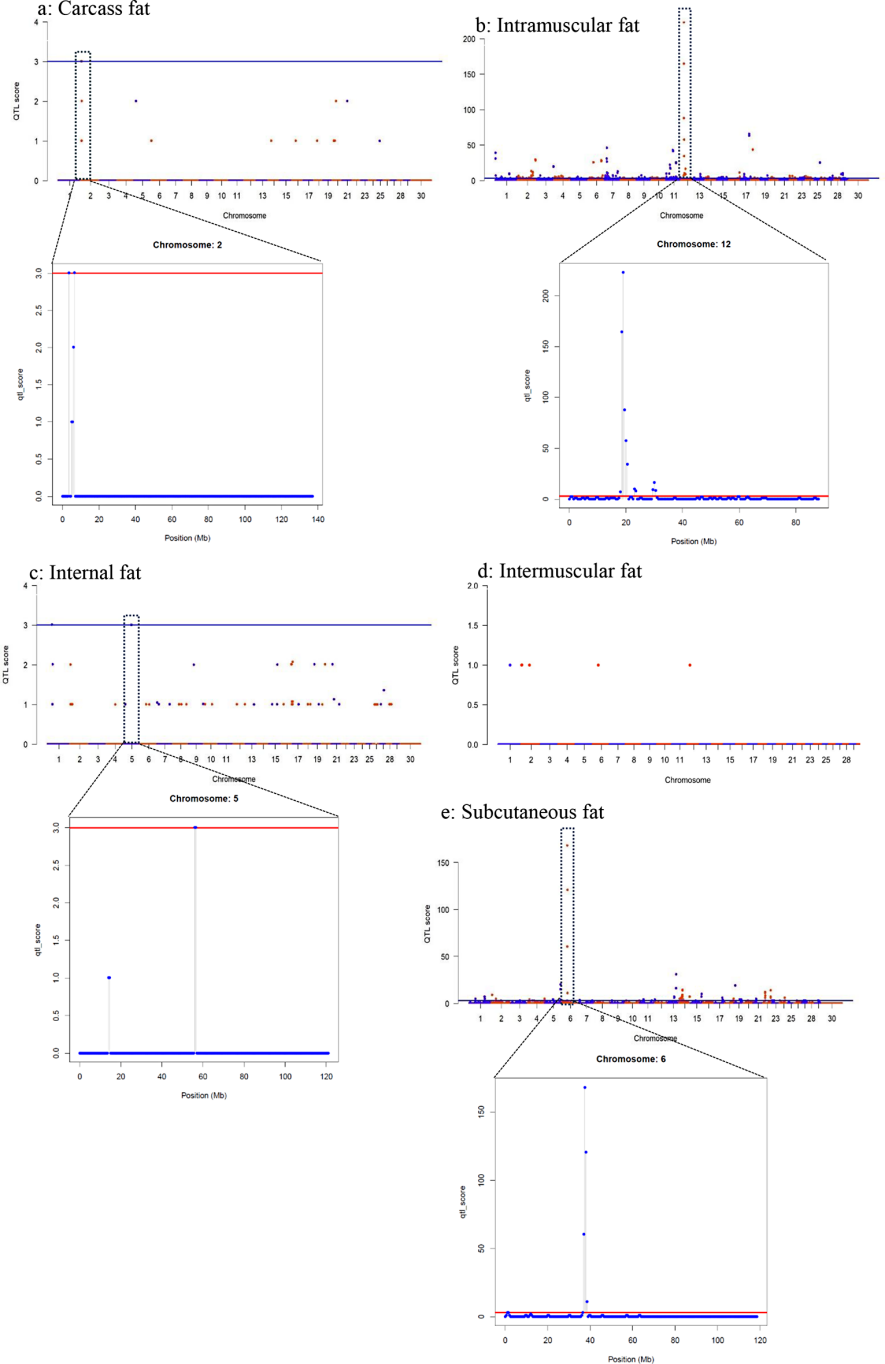
Manhattan plots of Meta-QTL for the fat trait groups. Fat trait groups include (**a**) Carcass fat, (**b**) Intramuscular fat, (**c**) Internal fat, (**d**) Intermuscular fat, and (**e**) Subcutaneous fat. Each dot represents one Meta-QTL position associated with a fat related trait. For each trait group, the first Manhattan plot is genome-wide with the most significant Meta-QTL highlighted in the dashed box. The second Manhattan plot for each trait group illustrates the chromosome with most significant Meta-QTL. The blue line is the score threshold of 3 for the genome-wide plots, while red line is the threshold of 3 for the chromosome-wide plots. X-axis represents the chromosomal positions and Y-axis represents Meta-QTL score against the corresponding map positions. Blue dots on Manhattan plots represent QTL or SNPs on the odd numbered chromosomes, while red dots represent QTL or SNPs on the even numbered chromosomes. The candidate genes for the most significant Meta-QTL are provided in the supplementary tables

The meta-analysis of the fat traits found 428 Meta-QTL in a total 216 regions (Table 3).Based on the 31 associations for carcass fat traits in the database records, 2 Meta-QTL regions were found on BTA2 at 3.5 Mb and 6.5 Mb with thresholds over 3.0 (Fig. 2a; Table 2). For the intramuscular fat traits, there were 315 Meta-QTL in 158 regions of the bovine genome based on 2186 reported associations (Table 2). The top ranked Meta-QTL for intramuscular fat were found on BTA12 in 3 regions (18-20.5 Mb, 23-23.5 Mb and 29.5–30.5 Mb) (Fig. 2b; Table 2). The meta-analysis of the internal fat traits for cattle found 4 Meta-QTL, based on 85 reported associations, located in 2 regions at 31.0-31.5 Mb on BTA1 and at 56.0 -56.5 Mb on BTA5 (Fig. 2c; Table 2). There were no Meta-QTL identified in the meta-analysis of the intermuscular fat traits (Fig. 2d; Table 2). However, the results reported on intermuscular fat traits were limited to 2 records with only 6 reported associations (Table 2). From the meta-analysis of the subcutaneous fat traits, 107 Meta-QTL located in 55 regions were found in the cattle genome, based on 887 reported associations (Table 2; Fig. 2e). The top ranked QTL for subcutaneous fat were located on BTA6 in 2 regions (1.0-1.5 Mb and 36.5–38.5 Mb) (Fig. 2e; Table 2).

### Candidate genes in top ranked Meta-QTL

Based on the location of the Meta-QTL regions (Table 3), genes within those regions were identified for each fat trait group using the bovine ARS-UCD 1.2 reference genome data available on Ensembl (https://asia.ensembl.org/index.html). The number of genes associated with each trait varied, as the size of the Meta-QTL regions differed among the trait groups. There were 20 genes in 2 Meta-QTL regions found for carcass fat and 84 genes in 2 Meta-QTL regions for internal fat (Table 3). In contrast, there were 1336 genes in 55 Meta-QTL regions for subcutaneous fat and 3853 genes in 158 Meta-QTL regions for intramuscular fat (Table 3). Due to absence of Meta-QTL regions for the intermuscular fat traits, no associated genes could be identified for this particular fat depot.

To identify the candidate genes which are most likely to be controlling the fat depots, the genes within these Meta-QTL regions can screened for their function using two databases, Gene Cards and DAVID. As examples, the genes in the Meta-QTL for carcass fat on BTA2 (Supplementary Table S2) and for intramuscular fat on BTA12 (Supplementary Table S3) were evaluated for their potential roles in fat deposition. On BTA2, which harbours two Meta-QTL regions for carcass fat, 20 genes were found in the two regions using the Ensembl database with one candidate gene, *MSTN*, identified based on its function (Table 4). For intramuscular fat, there were 104 genes in the Meta-QTL on BTA 12 (Table 4) of which 7 candidate genes (*RCBTB1*, *RCBTB2*, *SPRYD7*,*LHFPL6*,* MED4*,*MEDAG*, and *ALOX5AP*) were identified as being most likely to control fat deposition (Table 4 and Supplementary Table S4).

**Table 4:** Physiological function of candidate genes in Meta-QTL for different fat trait groups

Physiological category	Trait group	QTL location	Gene symbol	Gene name	Gene location (BTA: bp)	Reference
adipogenesis	Internal & Subcutaneous	BTA5, 56.5 Mb	*STAT2*	signal transducer and activator of transcription 2	12:56,341,597 − 56,360,203	Jiang et al., [33]; Xu et al., [32]
adipogenesis	Internal & Subcutaneous	BTA5, 56 Mb	*STAT6*	signal transducer and activator of transcription 6	12:57,095,408 − 57,132,139	Szanto et al., [34]; Baeza et al., [36]; Rincon et al., [37]
adipogenesis	Internal & Subcutaneous	BTA5, 56 Mb	*DDIT3*	DNA damage inducible transcript 3	12:57,516,588 − 57,521,737	Wei et al., [26]
adipogenesis, lipogenesis	IMF	BTA12, 18 Mb	*MED4*	mediator complex subunit 4	13:48,053,323 − 48,095,131	Han et al., [27]; Zhang et al., [23]
adipogenesis, lipogenesis	IMF & Subcutaneous	BTA6, 37 Mb	*MED28*	mediator complex subunit 28	4:17,614,641 − 17,634,105	Chou et al., [30]
adipogenesis, lipogenesis	IMF	BTA12, 29.5 Mb	*MEDAG*	mesenteric estrogen dependent adiposis	13:30,906,271 − 30,925,572	Zhang et al., [23]; Tizioto et al., [40]
adipogenesis, lipogenesis	Internal & Subcutaneous	BTA5, 56 Mb	*LRP1*	LDL receptor related protein 1	12:57,128,483 − 57,213,361	Masson et al., [29]
myogenesis	Internal & Subcutaneous	BTA5, 56 Mb	*ARHGEF25*	Rho Guanine Nucleotide Exchange Factor 25	12:57,610,116 − 57,617,245	Bryan et al., [31]; Li et al., [46]
myogenesis	Carcass fat	BTA2, 6.5 Mb	*MSTN*	myostatin	2: 6,278,864-6,285,486	Bittante et al., [42]; Fiems et al., [43]
muscle growth	Internal & Subcutaneous	BTA5, 56.5 Mb	*NACA*	nascent polypeptide associated complex subunit alpha	12:56,712,305 − 56,731,628	Bryan et al., [31]; Li et al., [46]
myogenesis	Internal & Subcutaneous	BTA5, 56 Mb	*TSPAN31*	tetraspanin 31	12:57,738,013–57,750,219	Li et al., [21]; Meienberg et al., [44]
lipoma	IMF	BTA12, 19 Mb	*SPRYD7*	SPRY domain containing 7	13:49,912,702 − 49,936,490	Grigoriadis et al., [51]; Montero-Calle et al., [50]
lipoma	IMF	BTA12, 23 Mb	*LHFPL6*	LHFPL tetraspan subfamily member 6	13:39,209,116 − 39,603,528	Grigoriadis et al., [51]; Montero-Calle et al., [50]
lipoma, lipomatosis	IMF	BTA12, 18 Mb	*RCBTB2*	RCC1 and BTB domain containing protein 2	13:48,488,963 − 48,535,895	by Creytens et al., [49]
liposarcoma	IMF	BTA12, 19 Mb	*RCBTB1*	RCC1 and BTB domain containing protein 1	13:49,531,946 − 49,585,558	Lee et al., [21]
liposarcoma	Internal & Subcutaneous	BTA5, 56 Mb	*CDK4*	cyclin dependent kinase 4	12:57,747,727 − 57,756,013	Wu et al., [17]2022
lipodystrophy	Internal & Subcutaneous	BTA5, 56.5 Mb	*PRIM1*	RCC1 and BTB domain containing protein 2	12:56,730,438 − 56,752,374	Parry et al., [48]

IMF = intramuscular fat

### Candidate genes in overlapping QTL

The overlapping QTL are of particular interest as they may represent genes that control the adipogenesis of more than one fat depot. For the four fat trait groups with Meta-QTL, there were 39 Meta-QTL in 16 Meta-QTL regions which overlapped between the trait groups, and these Meta-QTL contained 311 genes (Tables 3 and 5). Of the overlapping Meta-QTL, 14 of the 16 Meta-QTL were associated with IMF and subcutaneous fat. There were only 2 of the 16 overlapping Meta-QTL regions that were associated with other fat depots, a Meta-QTL region on BTA2 at 3.0-3.5 Mb associated with carcass fat, IMF and subcutaneous fat and a Meta-QTL region on BTA5 at 56.0-56.5 Mb associated with internal fat and subcutaneous fat.

**Table 5:** Overlapping Meta-QTL for different fat trait groups

Chromosome (BTA)	Position (Mb)	QTL score	Meta-QTL ID	Trait
1	44.5	5.08	90	IMF
1	44.5	5.02	90	Subcutaneous fat
1	45.0	4.01	91	IMF
2	3.0	3.03	327	IMF
2	3.5	3.01	328	Carcass fat
2	3.5	4.03	328	IMF
2	3.5	9.02	328	Subcutaneous fat
4	28.5	3.00	898	Subcutaneous fat
4	29.0	3.54	899	IMF
4	29.0	3.00	899	Subcutaneous fat
4	29.5	3.54	900	IMF
5	56.0	3.00	1196	Internal fat
5	56.0	4.01	1196	Subcutaneous fat
5	56.5	3.00	1197	Internal fat
5	56.5	4.01	1197	Subcutaneous fat
6	36.5	25.48	1400	IMF
6	36.5	3.01	1400	Subcutaneous fat
6	37.0	25.48	1401	IMF
6	37.0	60.48	1401	Subcutaneous fat
6	37.5	168.22	1402	Subcutaneous fat
6	38.0	120.74	1403	Subcutaneous fat
6	38.5	11.00	1404	Subcutaneous fat
7	5.5	5.06	1576	IMF
7	6.0	5.06	1577	IMF
7	6.5	3.63	1578	Subcutaneous fat
13	48.0	9.01	2929	IMF
13	48.0	7.00	2929	Subcutaneous fat
13	48.5	6.00	2930	IMF
13	48.5	6.00	2930	Subcutaneous fat
14	3.0	4.03	3008	IMF
14	3.0	5.01	3008	Subcutaneous fat
14	3.5	4.03	3009	IMF
14	4.0	3.01	3010	IMF
14	17.0	5.01	3036	IMF
14	17.0	3.01	3036	Subcutaneous fat
14	17.5	3.00	3037	IMF
14	17.5	3.01	3037	Subcutaneous fat
14	23.5	5.01	3049	IMF
14	23.5	9.02	3049	Subcutaneous fat
14	24.0	3.00	3050	IMF
14	24.0	7.01	3050	Subcutaneous fat
15	33.5	3.72	3236	IMF
15	34.0	4.78	3237	IMF
15	34.0	3.13	3237	Subcutaneous fat
19	50.0	7.01	3890	IMF
19	50.0	4.00	3890	Subcutaneous fat
20	32.0	3.07	3983	IMF
20	32.0	3.01	3983	Subcutaneous fat
20	32.5	3.07	3984	IMF
20	32.5	3.01	3984	Subcutaneous fat
23	13.0	3.74	4357	IMF
23	13.5	3.61	4358	IMF
23	14.0	3.01	4359	Subcutaneous fat
23	14.5	3.01	4360	Subcutaneous fat
24	2.0	5.71	4442	IMF
24	2.0	3.00	4442	Subcutaneous fat
24	2.5	3.00	4443	IMF
24	2.5	3.00	4443	Subcutaneous fat
24	38.0	4.12	4514	IMF
24	38.0	6.01	4514	Subcutaneous fat
24	38.5	3.01	4515	Subcutaneous fat

IMF = intramuscular fat

These 2 unusual overlapping Meta-QTL regions and the top ranking overlapping Meta-QTL for intramuscular fat and subcutaneous fat on BTA6 at 36.5–38.5 Mb were selected for candidate gene screening as they were representative of the four fat trait groups. There were 4 genes within the overlapping Meta-QTL regions on BTA2 at 3.0-3.5 Mb for the carcass, intramuscular and subcutaneous fat traits (Supplementary Table S5). Three of these loci are predicted genes without extensive homology to any known genes, and hence, their functions are unknown. Therefore, no candidate genes were identified for this QTL region. However, there were 104 genes found within the overlapping Meta-QTL regions for intramuscular, subcutaneous and internal fat traits on BTA5 and BTA6 (Supplementary Table S5), of which there were 15 and 3 genes identified, respectively, as candidates based on their function (*STAT2*, *STAT6*, *DDIT3*, *LRP1*, *ARHGEF25*, *NACA*, *TSPAN31*, *PRIM1*, *CDK4*, *PTGES3*, *PIP4K2C*, *B4GALNT1*, *CYP27B1*, *RDH16*, *HSD17B6*, *PPM1K*, *FAM13A*, and *MED28*; Table 4 and Supplementary Table S4).

## Discussion

In this study, a meta-analysis was conducted to identify the Meta-QTL regions and corresponding genes influencing fat deposition in cattle, specifically intramuscular fat, intermuscular fat, carcass fat, internal fat and subcutaneous fat as these are of importance to beef industry. The meta-analysis allowed the identification of bovine fat-related QTL that were consistently observed across multiple research projects using different statistical methods, measurements and breeds of cattle. Given the heterogeneity in effect size reporting across studies, we implemented a P-value based weighting system for QTLs to ensure consistent comparison. Thus, the meta-analysis, based on data from the cattle Animal QTLdb, found more precise QTL positions by leveraging the additional information provided by multiple studies compared to the individual studies. In addition, this analysis identified overlapping meta-QTL to address the question if there are genes with pleiotropic effects on fat depots. However, it should be noted that consensus estimates may not fully capture population-specific variants, potentially overlooking unique genetic features within individual herds or breeds.

Except for the intermuscular fat trait group which had limited records (*n* = 6), all fat trait groups had Meta-QTL regions identified. The number of Meta-QTL were different for different trait groups and varied greatly. For example, the intramuscular fat trait and subcutaneous fat trait groups had the most Meta-QTL, whereas the carcass fat trait and internal fat group had the least (Table 3). The difference in the number of Meta-QTL between the trait groups was reflected in the different number of publications for these traits (Table 1) and suggests that the larger number of Meta-QTL for intramuscular fat and subcutaneous fat is a result of the greater number of observed significant associations rather than a greater number of genes controlling these fat depots.

These Meta-QTL are likely to contain genetic loci of sizeable effect and alleles that are not likely to be breed specific since they were found in multiple studies. However, the resolution of the QTL was not always sufficient to distinguish between several individual Meta-QTL of small effect and a major Meta-QTL of large effect. Meta-QTL were often found in different regions of a single chromosome for the fat trait groups, but the exact number of QTL was not always obvious. As an example, for carcass fat, the Meta-QTL regions were on the same chromosome in nearby regions, namely at 3.5 Mb and 6.5 Mb on BTA2 (Fig. 2a; Table 2), and therefore, these may represent either a single genetic locus of large effect or two genetic loci of smaller effect. The Meta-QTL regions for intramuscular fat on BTA12 were also dispersed at 19 Mb, 23 Mb and 30 Mb (Fig. 2b; Table 2), and given the distances, may represent 3 loci or 2 loci at ~ 20 Mb and at ~ 30 Mb. In contrast, the 2 Meta-QTL regions for subcutaneous fat on BTA1 were further apart, at 1 Mb and 37 Mb (Fig. 2e), and consequently, are very likely to represent 2 different QTL.

The identification of the Meta-QTL regions also showed that several QTL overlapped on the same chromosome for different fat traits (Table 5). As examples, the Meta-QTL for intramuscular fat, carcass fat and subcutaneous fat overlapped on BTA2 and the Meta-QTL for internal fat and subcutaneous fat overlapped on BTA5. The other 14 overlapping Meta-QTL were all associated with subcutaneous fat and intramuscular fat. This is likely a reflection of the larger number of Meta-QTL discovered for these two fat depots.

Other authors have previously reported the similar results where one gene or one QTL was significantly associated with more than one fat depot in cattle. For example, Gutiérrez-Gil et al. found QTL for subcutaneous fat content, fat depth at the level of the third lumbar vertebra and kidney knob and channel fat were in the same region on BTA6 [19]. Similarly, in a Nellore cattle population, both intramuscular fat content and backfat thickness were significantly associated with the same QTL on BTA7 [20]. In some instances, even candidate genes in overlapping QTL have been found to control different fat traits. For example, in a study which investigated the mutations in the regulatory region of the ubiquinol-cytochrome c reductase core 1 (*UQCRC1*) gene, the results revealed that marbling fat and back fat were controlled by two functional mutations resulting in abnormal energy metabolism in the muscles of cattle [21].

This implies the pleiotropy of QTL and suggests that the candidate genes in shared regions may play pivotal roles simultaneously in adipogenesis or lipid metabolism of different fat depots [22, 23]. Overlapping QTL may perhaps even control unrelated traits. Lee et al. found QTL for different traits (namely, marbling, back-fat thickness and carcass weight) in a small region of BTA 14 [21]. This may occur if one trait is correlated physiologically with another trait, in this instance, the inverse relationship between muscularity and fatness in cattle. The overlapping QTL could be also a result of linkage disequilibrium (LD) between the causative variants [22]. Most of the Meta-QTL that overlapped were for intramuscular fat and subcutaneous fat, and this would suggest that some of the same gene variants are controlling fat deposition in both depots. However, the fat traits appear to be mainly controlled by different Meta-QTL on different chromosomal regions in beef cattle as only 39 of the 428 significant 1 Mb windows overlapped between trait groups.

In contrast, for a given fat depot, overlapping QTL for different measurements of the same fat depot are expected. In beef cattle, fat depots are often measured by different methods or described into different terms. Consequently, for this meta-analysis study, different fat trait phenotypes were assigned to one trait group to identify the corresponding QTL and SNPs. For example, traits such as fat area to ribeye area ratio, intramuscular fat, marbling score and meat fat content were classified into the intramuscular fat group for the meta-analysis. Given the correlations between the different measures of a given depot, the same QTL or genes should be found. As an example, in the study conducted by Lee et al., intramuscular fat was measured as marbling score and IMF content and both traits were correlated with transcripts from LOC614744 on BTA14 in Hanwoo cattle [21].

There were 20, 84, 1336 and 3853 candidate genes predicted from Ensembl for the carcass, internal, subcutaneous and intramuscular fat groups, respectively. As there were too few genes for carcass fat and internal fat and too many genes for subcutaneous fat and intramuscular fat, a gene network analysis based on these positional candidates did not lead to any useful pathways. Therefore, the positional candidate genes in the most significant Meta-QTL for each fat trait group were examined for their physiological functions. Of the genes in the Meta-QTL regions on BTA2, BTA5, BTA6 and BTA12, seven candidate genes were identified based on their roles in the adipogenesis of multiple fat depots and regulating lipid homeostasis and metabolism (*STAT2*, *STAT6*, *DDIT3*, *MED4*, *MED28*,*MEDAG*, and *LRP1*) [24–31]. STAT2 (signal transducer and activator of transcription 2) and JAK2 (Janus kinase 2) are part of the JAK2/STAT2 pathway which is activated by fatty acid binding protein 4 (FABP4). The CD36-mediated lipoprotein entry and ABCA1-dependent lipid efflux are inhibited by FABP4, which lowers intracellular lipid content by affecting lipid metabolism [32, 33]. STAT6 (signal Transducer and Activator of Transcription 6) promotes lipid accumulation by inducing the switch from glycolysis towards fatty acid oxidation. In this process, STAT6 interacts with PPAR gamma (the peroxisome proliferator-activated receptor gamma) to bind the promoter of the *FABP4* gene to activate lipid transcription in adipose metabolism [34]. Other genes act through different pathways. DDIT3 (DNA damage inducible transcript 3) protein has been implicated in adipogenesis by negatively regulating C/EBP-induced transcription, while MED4 (mediator complex subunit 4) acts as transcriptional factor in the regulation of white adipocyte differentiation and regulates lipid metabolism via PPAR alpha [26, 27]. MED28 (mediator complex subunit 28) promotes lipid accumulation with higher expression levels of sterol regulatory element binding protein 1 gene (*SREBP1*) through MED28 /mTOR (mammalian target of rapamycin) signalling [30]. Similarly, LRP1 (low-density lipoprotein receptor-related protein 1) is involved in the process of differentiation during adipogenesis, and the silencing of the *LRP1* gene in preadipocytes can significantly inhibit the expression of differentiation markers including *PPAR gamma*, *HSL* (hormone-sensitive lipase) and *aP2* (adipocyte fatty acid binding protein) [29]. In the study of female mice, the MEDAG protein (mesenteric estrogen dependent adipogenesis) was found to enhance preadipocyte differentiation and increase the lipid accumulation of mature adipocytes [35].

The association between these candidate genes and fat traits in cattle and other species has been observed by other researchers. For instance, *STAT6* on chromosome 5 has been previously found to be associated with fat traits (back fat and intramuscular fat percentage) in different cattle breeds [36, 37]. A significant relationship between *MED4* and fat traits (fat bandwidth, skin fat thickness, and abdominal fat weight) was found by Zhang et al. in a Gushi-Anka F2 chicken population [38]. The *MED4* gene was observed to have a higher transcriptional level in Jinghuang chicken samples with high triglyceride content [39]. In addition, Wei et al. found that the *MED4* gene had significantly higher expression on the late period of preadipocyte differentiation in subcutaneous fat tissue and promoted cellular lipid accumulation in Chinese Red Steppe calves [26]. A relationship between MEDAG and backfat thickness and its role in adipogenesis was also observed in pig lines [40]. In genetically obese Leprdb/db mice, the deletion of *DDIT3* increased body fat mass but did not change the adipocyte cell size. The negative correlation between *eIF2α* (the upstream target of DDIT3) and adipocyte differentiation was also shown in mouse embryonic fibroblasts and genetically engineered mice [27].

The negative correlation between muscle and fat accumulation [41] suggest that genes responsible for myogenesis should be considered in the search for fat deposition candidate genes as their function in muscle growth may have potential biological roles in adipogenesis. For instance, previous studies have shown that there is not only an increase in carcass weight and higher lean meat yield in cattle carrying *MSTN* mutations, but also decreased fat deposition in the skeletal muscles [42, 43]. As another example of genes affecting myogenesis and adipogenesis, ARHGEF25 (Rho Guanine Nucleotide Exchange Factor 25) promotes myogenesis of C2C12 cells but also inhibits insulin-induced adipogenesis in 3T3L1 preadipocytes [31]. Other genes involved in myogenesis that may affect adipogenesis include *TSPAN31* (tetraspanin 31), which is considered a genetic marker for cell growth and muscling [44, 45], and *NACA* (nascent polypeptide associated complex subunit alpha), which has been found to control myogenesis and have the relationship with myofibril organization [46].

Intramuscular fat deposition in humans is a disorder in adipose homeostasis [47]. Intramuscular fat deposition occurs in diseases such as lipoma and lipodystrophy in humans [48, 49] and this fat deposition may be controlled by the same genes and pathways as intramuscular fat deposition in cattle. Therefore, genes known to be related to lipomas were also considered as potential candidate genes, and included *SPRYD7*, *LHFPL6*, *RCBTB2*, *RCBTB1*, and *CDK4* [50–54]. In addition, genes affecting lipodystrophy, which involves the abnormal distribution of fat in patients, should be taken in consideration as these genes are implicated in fat deposition and/or fat loss. For example, individuals with *PRIM1* (*RCC1 and BTB domain containing protein 2*) deficiency have reduced subcutaneous fat deposition [48]. Lastly, although genes related to lipid metabolism are not known to be directly related to fat deposition, genes involved in lipid metabolism may affect adipose function. These potential candidate genes (*ALOX5AP*, *PTGES3*, *PIP4K2C*, *B4GALNT1*, *CYP27B1*, *PPM1K*,*FAM13A*,*ABCG2*,*RDH16*, and *HSD17B6)* could be explored further if variants in the other candidate genes are not associated with the fat deposition traits.

In conclusion, using the cattle Animal QTLdb and Ensembl databases, we conducted a meta-analysis to identify the related genomic regions for regulatory DNA variants and genes associated with fat depots in cattle, including carcass fat, intramuscular fat, internal fat, intermuscular fat and subcutaneous fat. Compared to individual experiments, meta-analyses are not only time-saving and efficient, but also can be repeated easily following updates of the databases. The identified Meta-QTL regions are beneficial for the prediction of candidate genes for causative variant discovery. However, because less than 10% of the fat-related Meta-QTL overlapped, most causative variants are not likely to be pleiotropic with large effects in more than one fat depot. For further exploration, a pathway analysis of the candidate genes regulating fat metabolism in different cattle fat depots may help to better understand why genes may or may not affect more than one trait or more than one physiological pathway such as myogenesis versus adipogenesis.

## Electronic supplementary material

Below is the link to the electronic supplementary material.

Supplementary Material 1

## Data Availability

All data analysed during this study are included in this published article are available at https://www.animalgenome.org/QTLdb/doc/download.

## Abbreviations

QTL: Quantitative Trait Locus
Meta-QTL: Meta-Quantitative Trait Locus
LD: Linkage Disequilibrium
Cattle QTLdb: Cattle Quantitative Trait Locus (QTL) Database
SNP: Single Nucleotide Polymorphism
cM: Centimorgan
DNA: Deoxyribonucleic Acid
DAVID: Database for Annotation, Visualization and Integrated Discovery
BTA: Bos Taurus Autosome
IMF: Intramuscular Fat
