# Supplementary material for: Meta-assembly of genomic associations to identify cattle fat depot candidate genes and pleiotropic effects
Source: BMC Genomics. 2024 Dec 24;25:1242. doi: 10.1186/s12864-024-11159-4 (PMC11667861; doi:10.1186/s12864-024-11159-4)
Supplement: Supplementary file 1 — Supplementary Material 1 [file 12864_2024_11159_MOESM1_ESM.pdf]

Supplementary Table S1. Summary of Meta-QTL for the fat trait groups.

Chromosome (BTA)	Position (Mb)	QTL score	Meta-QTL ID	Trait
1	1.0	31.02	3	IMF
1	1.5	39.02	4	IMF
1	2.0	7.00	5	IMF
1	9.0	3.07	19	IMF
1	9.5	3.07	20	IMF
1	31.0	3.01	63	Internal fat
1	31.5	3.01	64	Internal fat
1	34.0	3.42	69	IMF
1	43.0	3.56	87	IMF
1	44.5	5.08	90	IMF
1	44.5	5.02	90	Subcutaneous fat
1	45.0	4.01	91	IMF
1	47.5	3.14	96	IMF
1	98.5	9.78	198	IMF
1	99.0	8.91	199	IMF
1	106.0	3.26	213	IMF
1	106.5	3.26	214	IMF
1	108.5	7.01	218	Subcutaneous fat
1	109.0	7.01	219	Subcutaneous fat
1	110.0	5.01	221	Subcutaneous fat
1	110.5	5.01	222	Subcutaneous fat
1	153.0	3.84	307	IMF
1	153.5	4.86	308	IMF
2	3.0	3.03	327	IMF
2	3.5	3.01	328	Carcass fat
2	3.5	4.03	328	IMF
2	3.5	9.02	328	Subcutaneous fat
2	4.5	6.02	330	IMF
2	5.0	6.46	331	IMF
2	5.5	3.11	332	IMF
2	6.5	3.01	334	Carcass fat
2	7.5	3.34	336	IMF
2	15.5	4.11	352	IMF
2	16.0	4.11	353	IMF
2	30.0	5.00	381	Subcutaneous fat
2	30.5	3.00	382	Subcutaneous fat
2	34.0	3.39	389	IMF
2	34.5	4.42	390	IMF
2	61.0	3.01	443	IMF
2	61.5	3.01	444	IMF
2	65.0	3.00	451	Subcutaneous fat
2	65.5	3.00	452	Subcutaneous fat

2	79.5	3.01	480	IMF
2	80.0	4.01	481	IMF
2	81.5	3.02	484	IMF
2	82.0	3.02	485	IMF
2	91.5	3.33	504	IMF
2	92.0	3.33	505	IMF
2	94.0	3.64	509	IMF
2	96.0	7.01	513	IMF
2	96.5	13.24	514	IMF
2	97.0	6.23	515	IMF
2	106.0	11.76	533	IMF
2	106.5	10.75	534	IMF
2	107.0	7.29	535	IMF
2	107.5	7.29	536	IMF
2	125.5	29.59	572	IMF
2	126.0	28.28	573	IMF
3	8.5	3.18	613	IMF
3	15.5	3.06	627	IMF
3	16.0	3.06	628	IMF
3	23.0	7.01	642	IMF
3	50.5	3.04	697	IMF
3	51.0	3.04	698	IMF
3	64.0	3.00	724	Subcutaneous fat
3	81.5	3.37	759	IMF
3	111.5	3.01	819	IMF
3	116.5	19.5	829	IMF
3	117.0	19.5	830	IMF
4	5.0	4.08	851	IMF
4	5.5	5.08	852	IMF
4	10.5	4.01	862	Subcutaneous fat
4	15.0	4.47	871	IMF
4	28.5	3.00	898	Subcutaneous fat
4	29.0	3.54	899	IMF
4	29.0	3.00	899	Subcutaneous fat
4	29.5	3.54	900	IMF
4	31.0	5.01	903	IMF
4	31.5	5.01	904	IMF
4	36.5	3.01	914	IMF
4	43.0	3.01	927	Subcutaneous fat
4	44.0	3.09	929	IMF
4	50.5	6.49	942	IMF
4	51.0	6.49	943	IMF
4	59.0	5.49	959	IMF
4	59.5	4.51	960	IMF
4	60.0	3.87	961	IMF
4	105.5	3.38	1052	IMF

4	106.0	3.38	1053	IMF
4	112.5	3.24	1066	IMF
4	113.0	3.24	1067	IMF
4	117.0	5.01	1075	Subcutaneous fat
4	117.5	4.00	1076	Subcutaneous fat
5	40.0	3.73	1164	IMF
5	47.0	3.12	1178	IMF
5	47.5	5.16	1179	IMF
5	48.0	3.04	1180	IMF
5	56.0	3.00	1196	Internal fat
5	56.0	4.01	1196	Subcutaneous fat
5	56.5	3.00	1197	Internal fat
5	56.5	4.01	1197	Subcutaneous fat
5	72.0	3.33	1228	IMF
5	109.0	4.00	1302	Subcutaneous fat
5	109.5	20.01	1303	Subcutaneous fat
5	110.0	15.01	1304	Subcutaneous fat
6	1.0	3.00	1329	Subcutaneous fat
6	1.5	3.00	1330	Subcutaneous fat
6	9.5	3.84	1346	IMF
6	22.5	4.04	1372	IMF
6	23.0	3.00	1373	IMF
6	27.0	4.11	1381	IMF
6	27.5	3.13	1382	IMF
6	36.5	25.48	1400	IMF
6	36.5	3.01	1400	Subcutaneous fat
6	37.0	25.48	1401	IMF
6	37.0	60.48	1401	Subcutaneous fat
6	37.5	168.22	1402	Subcutaneous fat
6	38.0	120.74	1403	Subcutaneous fat
6	38.5	11.00	1404	Subcutaneous fat
6	63.0	3.09	1453	IMF
6	72.5	3.67	1472	IMF
6	93.0	28.32	1513	IMF
6	93.5	27.32	1514	IMF
6	95.5	4.24	1518	IMF
6	96.0	4.24	1519	IMF
6	102.5	6.97	1532	IMF
6	103.0	6.97	1533	IMF
7	2.5	5.44	1570	IMF
7	3.0	6.80	1571	IMF
7	5.5	5.06	1576	IMF
7	6.0	5.06	1577	IMF
7	6.5	3.63	1578	Subcutaneous fat
7	10.0	4.78	1585	IMF
7	10.5	4.78	1586	IMF

7	12.5	4.10	1590	IMF
7	13.5	30.68	1592	IMF
7	14.0	46.07	1593	IMF
7	14.5	26.44	1594	IMF
7	15.0	11.06	1595	IMF
7	15.5	4.16	1596	IMF
7	16.0	4.15	1597	IMF
7	17.5	3.02	1600	IMF
7	18.0	9.41	1601	IMF
7	18.5	10.41	1602	IMF
7	19.0	6.02	1603	IMF
7	19.5	3.01	1604	IMF
7	20.0	3.01	1605	IMF
7	20.5	3.01	1606	IMF
7	22.0	6.02	1609	IMF
7	22.5	6.02	1610	IMF
7	25.0	3.01	1615	IMF
7	25.5	3.01	1616	IMF
7	26.0	4.63	1617	IMF
7	26.5	4.63	1618	IMF
7	27.0	4.28	1619	IMF
7	27.5	4.28	1620	IMF
7	35.5	3.01	1636	IMF
7	36.0	3.01	1637	IMF
7	56.5	7.12	1678	IMF
7	57.0	6.10	1679	IMF
7	78.5	6.35	1722	IMF
7	79.0	6.35	1723	IMF
7	90.5	10.77	1746	IMF
7	91.0	12.77	1747	IMF
7	96.0	3.01	1757	Subcutaneous fat
7	96.5	3.01	1758	Subcutaneous fat
7	104.5	3.14	1774	IMF
7	105.0	4.17	1775	IMF
8	14.5	3.45	1818	IMF
8	23.0	3.01	1835	IMF
8	23.5	3.01	1836	IMF
8	24.0	3.01	1837	IMF
8	24.5	4.02	1838	IMF
8	84.0	3.30	1957	IMF
8	84.5	3.30	1958	IMF
9	10.5	3.00	2039	Subcutaneous fat
9	16.5	3.33	2051	IMF
9	17.0	4.37	2052	IMF
9	33.5	7.15	2085	IMF
9	34.0	7.15	2086	IMF

9	57.5	3.32	2133	IMF
9	58.0	4.33	2134	IMF
9	97.0	3.02	2212	IMF
9	97.5	3.53	2213	IMF
10	4.5	4.53	2240	IMF
10	6.5	3.01	2244	Subcutaneous fat
10	7.0	3.01	2245	Subcutaneous fat
10	13.5	3.05	2258	IMF
10	26.0	3.36	2283	IMF
10	39.5	4.05	2310	IMF
10	40.0	4.05	2311	IMF
10	41.0	4.09	2313	IMF
10	41.5	3.07	2314	IMF
10	75.0	3.02	2381	IMF
10	75.5	3.02	2382	IMF
10	84.5	3.02	2400	IMF
10	101.5	3.00	2434	Subcutaneous fat
11	1.0	9.60	2442	IMF
11	1.5	9.60	2443	IMF
11	3.0	3.03	2446	IMF
11	3.5	5.20	2447	IMF
11	22.0	9.59	2484	IMF
11	22.5	9.59	2485	IMF
11	24.5	4.00	2489	Subcutaneous fat
11	25.0	4.00	2490	Subcutaneous fat
11	27.5	4.21	2495	IMF
11	28.0	21.93	2496	IMF
11	28.5	17.72	2497	IMF
11	30.0	16.76	2500	IMF
11	30.5	16.76	2501	IMF
11	36.5	4.30	2513	IMF
11	37.0	4.30	2514	IMF
11	48.5	42.77	2537	IMF
11	49.0	41.29	2538	IMF
11	52.5	4.87	2545	IMF
11	53.0	4.87	2546	IMF
11	69.5	5.04	2579	IMF
11	70.0	5.04	2580	IMF
11	70.5	24.56	2581	IMF
11	71.0	25.6	2582	IMF
11	74.0	4.61	2588	IMF
11	84.0	3.01	2608	Subcutaneous fat
11	84.5	3.01	2609	Subcutaneous fat
11	98.0	4.55	2636	IMF
11	102.5	3.67	2645	IMF
11	105.0	4.00	2650	Subcutaneous fat

11	105.5	4.00	2651	Subcutaneous fat
12	18.0	7.10	2692	IMF
12	18.5	164.73	2693	IMF
12	19.0	223.31	2694	IMF
12	19.5	87.88	2695	IMF
12	20.0	57.53	2696	IMF
12	20.5	34.32	2697	IMF
12	23.0	10.00	2702	IMF
12	23.5	8.00	2703	IMF
12	29.5	9.29	2715	IMF
12	30.0	16.35	2716	IMF
12	30.5	8.47	2717	IMF
13	2.0	3.34	2837	IMF
13	2.5	3.34	2838	IMF
13	5.5	4.11	2844	IMF
13	48.0	9.01	2929	IMF
13	48.0	7.00	2929	Subcutaneous fat
13	48.5	6.00	2930	IMF
13	48.5	6.00	2930	Subcutaneous fat
13	54.0	3.99	2941	IMF
13	54.5	4.00	2942	IMF
13	61.5	16.00	2956	Subcutaneous fat
13	62.0	31.00	2957	Subcutaneous fat
13	62.5	16.00	2958	Subcutaneous fat
14	1.0	3.04	3004	IMF
14	3.0	4.03	3008	IMF
14	3.0	5.01	3008	Subcutaneous fat
14	3.5	4.03	3009	IMF
14	4.0	3.01	3010	IMF
14	8.0	4.00	3018	Subcutaneous fat
14	13.0	6.01	3028	Subcutaneous fat
14	13.5	6.01	3029	Subcutaneous fat
14	16.5	4.01	3035	IMF
14	17.0	5.01	3036	IMF
14	17.0	3.01	3036	Subcutaneous fat
14	17.5	3.00	3037	IMF
14	17.5	3.01	3037	Subcutaneous fat
14	19.5	5.01	3041	Subcutaneous fat
14	20.0	4.01	3042	Subcutaneous fat
14	20.5	8.02	3043	Subcutaneous fat
14	21.0	9.02	3044	Subcutaneous fat
14	22.5	13.6	3047	Subcutaneous fat
14	23.0	14.6	3048	Subcutaneous fat
14	23.5	5.01	3049	IMF
14	23.5	9.02	3049	Subcutaneous fat
14	24.0	3.00	3050	IMF

14	24.0	7.01	3050	Subcutaneous fat
14	28.5	4.01	3059	Subcutaneous fat
14	73.5	7.01	3149	Subcutaneous fat
14	74.0	7.01	3150	Subcutaneous fat
15	14.0	3.62	3197	IMF
15	14.5	3.62	3198	IMF
15	26.5	3.00	3222	IMF
15	27.0	4.03	3223	IMF
15	30.5	3.14	3230	IMF
15	31.0	3.14	3231	IMF
15	33.5	3.72	3236	IMF
15	34.0	4.78	3237	IMF
15	34.0	3.13	3237	Subcutaneous fat
15	37.0	3.84	3243	IMF
15	37.5	3.84	3244	IMF
15	38.5	4.03	3246	IMF
15	39.0	3.03	3247	IMF
15	41.0	3.77	3251	IMF
15	41.5	4.78	3252	IMF
15	45.5	3.23	3260	IMF
15	46.0	3.23	3261	IMF
15	67.5	4.70	3304	IMF
15	68.0	3.67	3305	IMF
15	75.5	7.00	3320	Subcutaneous fat
15	76.0	10.00	3321	Subcutaneous fat
16	59.5	3.04	3461	IMF
16	73.0	10.98	3488	IMF
16	73.5	11.02	3489	IMF
16	76.5	5.01	3495	IMF
16	77.0	4.00	3496	IMF
17	23.5	3.02	3554	IMF
17	24.0	5.24	3555	IMF
17	24.5	5.35	3556	IMF
17	25.0	8.85	3557	IMF
17	25.5	5.72	3558	IMF
17	37.5	3.42	3582	IMF
17	38.0	3.42	3583	IMF
17	55.5	3.00	3618	Subcutaneous fat
17	56.0	6.00	3619	Subcutaneous fat
17	56.5	4.00	3620	Subcutaneous fat
17	60.5	63.41	3628	IMF
17	61.0	65.66	3629	IMF
17	70.0	3.15	3647	IMF
17	70.5	4.96	3648	IMF
18	12.0	43.58	3680	IMF
18	12.5	43.58	3681	IMF

18	25.0	3.97	3706	IMF
18	25.5	3.97	3707	IMF
18	45.0	3.05	3746	IMF
18	45.5	3.05	3747	IMF
18	60.5	3.00	3777	Subcutaneous fat
18	61.0	5.00	3778	Subcutaneous fat
19	3.0	3.24	3796	IMF
19	3.5	3.24	3797	IMF
19	7.0	19.02	3804	Subcutaneous fat
19	7.5	19.02	3805	Subcutaneous fat
19	9.0	3.18	3808	IMF
19	39.0	4.01	3868	Subcutaneous fat
19	39.5	4.01	3869	Subcutaneous fat
19	47.5	4.01	3885	Subcutaneous fat
19	50.0	7.01	3890	IMF
19	50.0	4.00	3890	Subcutaneous fat
19	55.5	3.10	3901	IMF
20	12.5	4.11	3944	IMF
20	13.0	3.00	3945	IMF
20	13.5	3.00	3946	IMF
20	16.5	3.21	3952	IMF
20	17.0	4.56	3953	IMF
20	18.5	4.59	3956	IMF
20	19.0	4.59	3957	IMF
20	27.0	3.05	3973	IMF
20	32.0	3.07	3983	IMF
20	32.0	3.01	3983	Subcutaneous fat
20	32.5	3.07	3984	IMF
20	32.5	3.01	3984	Subcutaneous fat
21	19.0	4.00	4103	Subcutaneous fat
21	19.5	5.00	4104	Subcutaneous fat
21	20.0	3.00	4105	Subcutaneous fat
21	33.0	3.00	4131	IMF
21	33.5	3.00	4132	IMF
22	0.5	6.58	4208	IMF
22	1.0	4.05	4209	IMF
22	2.5	3.76	4212	IMF
22	3.0	3.76	4213	IMF
22	12.0	6.00	4231	Subcutaneous fat
22	12.5	11.00	4232	Subcutaneous fat
22	13.0	12.00	4233	Subcutaneous fat
22	13.5	8.01	4234	Subcutaneous fat
22	14.0	3.00	4235	Subcutaneous fat
22	20.5	6.24	4248	IMF
22	21.0	6.24	4249	IMF
22	38.5	4.02	4284	IMF

22	39.0	4.02	4285	IMF
22	52.0	7.00	4311	Subcutaneous fat
22	52.5	14.00	4312	Subcutaneous fat
22	53.0	6.00	4313	Subcutaneous fat
22	57.5	3.00	4322	Subcutaneous fat
22	58.0	3.00	4323	Subcutaneous fat
23	3.5	3.00	4338	Subcutaneous fat
23	11.5	3.00	4354	Subcutaneous fat
23	13.0	3.74	4357	IMF
23	13.5	3.61	4358	IMF
23	14.0	3.01	4359	Subcutaneous fat
23	14.5	3.01	4360	Subcutaneous fat
23	27.5	3.24	4386	IMF
23	28.0	3.72	4387	IMF
23	29.0	3.27	4389	IMF
23	33.5	3.93	4398	IMF
23	34.0	3.93	4399	IMF
23	45.5	5.20	4422	IMF
23	46.0	5.18	4423	IMF
23	46.5	4.12	4424	IMF
24	2.0	5.71	4442	IMF
24	2.0	3.00	4442	Subcutaneous fat
24	2.5	3.00	4443	IMF
24	2.5	3.00	4443	Subcutaneous fat
24	15.5	3.54	4469	IMF
24	16.0	4.57	4470	IMF
24	29.0	4.17	4496	IMF
24	29.5	4.17	4497	IMF
24	38.0	4.12	4514	IMF
24	38.0	6.01	4514	Subcutaneous fat
24	38.5	3.01	4515	Subcutaneous fat
25	6.0	3.10	4577	IMF
25	39.0	25.06	4643	IMF
25	39.5	25.06	4644	IMF
26	4.5	4.01	4661	IMF
26	5.0	4.01	4662	IMF
26	18.0	3.00	4688	Subcutaneous fat
26	28.5	3.50	4709	IMF
26	29.0	3.50	4710	IMF
27	13.0	4.13	4784	IMF
27	13.5	3.52	4785	IMF
27	15.5	3.00	4789	Subcutaneous fat
27	16.0	3.00	4790	Subcutaneous fat
27	25.5	3.00	4809	Subcutaneous fat
27	26.0	3.02	4810	IMF
27	33.5	6.52	4825	IMF

27	34.0	3.52	4826	IMF
28	0.5	3.27	4853	IMF
28	11.5	3.44	4875	IMF
28	12.0	3.44	4876	IMF
28	13.5	3.10	4879	IMF
28	34.0	3.07	4920	IMF
28	34.5	4.07	4921	IMF
28	42.5	3.02	4937	IMF
28	43.0	3.02	4938	IMF
29	1.5	9.13	4949	IMF
29	2.0	9.13	4950	IMF
29	12.5	7.88	4971	IMF
29	13.0	10.02	4972	IMF
29	17.0	3.36	4980	IMF
29	25.0	3.00	4996	Subcutaneous fat
29	25.5	3.00	4997	Subcutaneous fat
29	28.5	3.00	5003	IMF
29	29.0	3.00	5004	IMF

IMF = intramuscular fat

Supplementary Table S2. Genes in Meta-QTL for carcass fat on BTA 2 with candidate genes highlighted.

Ensembl gene id	Gene symbol	Gene name	position (Mb)	Meta-QTL ID
ENSBTAG00000049463	Predicted gene		3.5	328
ENSBTAG00000060094	Predicted gene		3.5	328
ENSBTAG00000065936	Predicted gene		3.5	328
ENSBTAG00000000492	*ASNSD1*	asparagine synthetase domain containing 1	6.5	334
ENSBTAG00000003002	*WDR75*	WD repeat domain 75	6.5	334
ENSBTAG00000007787	*HIBCH*	3-hydroxyisobutyryl-CoA hydrolase	6.5	334
ENSBTAG00000010498	*SLC40A1*	solute carrier family 40 member 1	6.5	334
ENSBTAG00000011808	*MSTN*	myostatin	6.5	334
ENSBTAG00000012554	*ANKAR*	ankyrin and armadillo repeat containing	6.5	334
ENSBTAG00000012557	*OSGEPL1*	O-sialoglycoprotein endopeptidase like 1	6.5	334
ENSBTAG00000012561	*ORMDL1*	ORMDL sphingolipid biosynthesis regulator 1	6.5	334
ENSBTAG00000018795	*PMS1*	PMS1 homolog 1, mismatch repair system component	6.5	334
ENSBTAG00000026994	*C2H2orf88*	chromosome 2 C2orf88 homolog	6.5	334
ENSBTAG00000056909	Predicted gene		6.5	334
ENSBTAG00000056954	Predicted gene		6.5	334
ENSBTAG00000061306	Predicted gene		6.5	334
ENSBTAG00000062554	Predicted gene		6.5	334
ENSBTAG00000062575	Predicted gene		6.5	334
ENSBTAG00000064129	Predicted gene		6.5	334
ENSBTAG00000070095	Predicted gene		6.5	334

Supplementary Table S3. Genes in Meta-QTL for intramuscular fat on BTA 12 with candidate genes highlighted.

Ensembl gene ID	Gene name	Description	Position (Mb)	Meta-QTL ID
ENSBTAG00000003109	*ITM2B*	integral membrane protein 2B	18.0	2692
ENSBTAG00000006640	*RB1*	RB transcriptional corepressor 1	18.0	2692
ENSBTAG00000006645	*LPAR6*	lysophosphatidic acid receptor 6	18.0	2692
ENSBTAG00000006647	*RCBTB2*	RCC1 and BTB domain containing protein 2	18.0	2692
ENSBTAG00000006759	*SUCLA2*	succinate-CoA ligase ADP-forming subunit beta	18.0	2692
ENSBTAG00000017508	*CYSLTR2*	cysteinyl leukotriene receptor 2	18.0	2692
ENSBTAG00000019501	*NUDT15*	nudix hydrolase 15	18.0	2692
ENSBTAG00000019502	*MED4*	mediator complex subunit 4	18.0	2692
ENSBTAG00000024964	Predicted gene		18.0	2692
ENSBTAG00000055459	Predicted gene		18.0	2692
ENSBTAG00000055793	Predicted gene		18.0	2692
ENSBTAG00000064647	*NUDT15*	Nudix Hydrolase 15	18.0	2692
ENSBTAG00000064733	Predicted gene		18.0	2692
ENSBTAG00000067973	Predicted gene		18.0	2692
ENSBTAG00000068226	Predicted gene		18.0	2692
ENSBTAG00000068507	Predicted gene		18.0	2692
ENSBTAG00000069081	Predicted gene		18.0	2692
ENSBTAG00000002271	*CDADC1*	cytidine and dCMP deaminase domain containing 1	18.5	2693
ENSBTAG00000010475	*MLNR*	motilin receptor	18.5	2693
ENSBTAG00000020681	*FNDC3A*	fibronectin type III domain containing 3A	18.5	2693
ENSBTAG00000034222	*CAB39L*	calcium binding protein 39 like	18.5	2693
ENSBTAG00000048524	Predicted gene		18.5	2693
ENSBTAG00000056732	Predicted gene		19.0	2694
ENSBTAG00000060386	Predicted gene		19.0	2694
ENSBTAG00000063116	Predicted gene		19.0	2694
ENSBTAG00000069989	Predicted gene		19.0	2694
ENSBTAG00000004850	*KPNA3*	karyopherin subunit alpha 3	19.0	2694
ENSBTAG00000007329	*SETDB2*	SET domain bifurcated histone lysine methyltransferase 2	19.0	2694
ENSBTAG00000008173	*TRIM13*	tripartite motif containing 13	19.0	2694
ENSBTAG00000008176	*KCNRG*	potassium channel regulator	19.0	2694
ENSBTAG00000013159	*PHF11*	PHD finger protein 11	19.0	2694
ENSBTAG00000016998	*ARL11*	ADP ribosylation factor like GTPase 11	19.0	2694
ENSBTAG00000018410	*EBPL*	EBP like	19.0	2694
ENSBTAG00000018829	*SPRYD7*	SPRY domain containing 7	19.0	2694
ENSBTAG00000034269	*RCBTB1*	RCC1 and BTB domain containing protein 1	19.0	2694
ENSBTAG00000047343	Predicted gene		19.0	2694
ENSBTAG00000049254	Predicted gene		19.0	2694

ENSBTAG00000057958	Predicted gene		19.0	2694
ENSBTAG00000069183	Predicted gene		19.0	2694
ENSBTAG00000069446	Predicted gene		19.5	2695
ENSBTAG00000029867	*MIR15A*	microRNA 15a	19.5	2695
ENSBTAG00000036389	*bta-mir-16a*	bta-mir-16a	19.5	2695
ENSBTAG00000055433	Predicted gene		19.5	2695
ENSBTAG00000058172	Predicted gene		19.5	2695
ENSBTAG00000059687	Predicted gene		19.5	2695
ENSBTAG00000062435	Predicted gene		19.5	2695
ENSBTAG00000063195	Predicted gene		19.5	2695
ENSBTAG00000067523	Predicted gene		19.5	2695
ENSBTAG00000007457	Predicted gene		20.0	2696
ENSBTAG00000020149	*RNASEH2B*	ribonuclease H2 subunit B	20.0	2696
ENSBTAG00000054472	*bta-mir-10177*	bta-mir-10177	20.0	2696
ENSBTAG00000055114	*DLEU7*	deleted in lymphocytic leukemia 7	20.0	2696
ENSBTAG00000057483	Predicted gene		20.0	2696
ENSBTAG00000064624	Predicted gene		20.0	2696
ENSBTAG00000065902	Predicted gene		20.0	2696
ENSBTAG00000066614	Predicted gene		20.0	2696
ENSBTAG00000067428	Predicted gene		20.5	2697
ENSBTAG00000002970	*INTS6*	integrator complex subunit 6	20.5	2697
ENSBTAG00000005284	*SERPINE3*	serpin family E member 3	20.5	2697
ENSBTAG00000046408	*FAM124A*	family with sequence similarity 124 member A	20.5	2697
ENSBTAG00000050638	Predicted gene		20.5	2697
ENSBTAG00000060393	Predicted gene		20.5	2697
ENSBTAG00000062865	Predicted gene		20.5	2697
ENSBTAG00000066825	Predicted gene		20.5	2697
ENSBTAG00000068013	*C13orf42*	chromosome 13 open reading frame 42	20.5	2697
ENSBTAG00000010949	*PROSER1*	proline and serine rich 1	23.0	2702
ENSBTAG00000010951	*NHLRC3*	NHL repeat containing 3	23.0	2702
ENSBTAG00000016635	*COG6*	component of oligomeric golgi complex 6	23.0	2702
ENSBTAG00000017032	*FREM2*	FRAS1 related extracellular matrix 2	23.0	2702
ENSBTAG00000018232	*STOML3*	stomatin like 3	23.0	2702
ENSBTAG00000034033	*LHFPL6*	LHFPL tetraspan subfamily member 6	23.0	2702
ENSBTAG00000042473	*SNORA48*	Small nucleolar RNA 48	23.0	2702
ENSBTAG00000063001	Predicted gene		23.5	2703
ENSBTAG00000051486	*UFM1*	ubiquitin fold modifier 1	23.5	2703
ENSBTAG00000053045	Predicted gene		23.5	2703
ENSBTAG00000055869	Predicted gene		23.5	2703
ENSBTAG00000062983	Predicted gene		23.5	2703
ENSBTAG00000065523	Predicted gene		23.5	2703
ENSBTAG00000066208	*U6*	U6 spliceosomal RNA	23.5	2703
ENSBTAG00000005012	*HSPH1*	heat shock protein family H (Hsp110) member 1	29.5	2715

ENSBTAG00000006771	*FRY*	FRY microtubule binding protein	29.5	2715
ENSBTAG00000008271	*MEDAG*	mesenteric estrogen dependent adiposis	29.5	2715
ENSBTAG00000015132	*RXFP2*	relaxin family peptide receptor 2	29.5	2715
ENSBTAG00000033386	*TEX26*	testis expressed 26	29.5	2715
ENSBTAG00000033412	*B3GLCT*	beta 3-glucosyltransferase	29.5	2715
ENSBTAG00000044236	*bta-mir-2299*	bta-mir-2299	29.5	2715
ENSBTAG00000056819	Predicted gene		29.5	2715
ENSBTAG00000061372	Predicted gene		29.5	2715
ENSBTAG00000061988	Predicted gene		29.5	2715
ENSBTAG00000063445	Predicted gene		29.5	2715
ENSBTAG00000063724	Predicted gene		29.5	2715
ENSBTAG00000064746	Predicted gene		29.5	2715
ENSBTAG00000065459	Predicted gene		29.5	2715
ENSBTAG00000001178	*USPL1*	ubiquitin specific peptidase like 1	30.0	2716
ENSBTAG00000009340	*KATNAL1*	katanin catalytic subunit A1 like 1	30.0	2716
ENSBTAG00000013201	*ALOX5AP*	arachidonate 5-lipoxygenase activating protein	30.0	2716
ENSBTAG00000018103	*HMGB1*	high mobility group box 1	30.0	2716
ENSBTAG00000056036	Predicted gene		30.0	2716
ENSBTAG00000059917	Predicted gene		30.0	2716
ENSBTAG00000066787	Predicted gene		30.0	2716
ENSBTAG00000069925	Predicted gene		30.0	2716
ENSBTAG00000012170	*UBL3*	ubiquitin like 3	30.5	2717
ENSBTAG00000018577	*SLC7A1*	solute carrier family 7 member 1	30.5	2717
ENSBTAG00000045239	*SNORA70*	Small nucleolar RNA SNORA70	30.5	2717

Supplementary Table S4. Candidate genes related to lipid metabolism in Meta-QTL regions for different fat trait groups.

Physiological category	Trait group	QTL location	Gene symbol	Gene name	Gene location (BTA: bp)
lipid metabolism	Internal & Subcutaneous	BTA5, 56.5 Mb	*PTGES3*	prostaglandin E synthase 3	12:56,663,341-56,688,408
lipid metabolism	Internal & Subcutaneous	BTA5, 56 Mb	*PIP4K2C*	phosphatidylinositol-5-phosphate 4-kinase type 2 gamma	12:57,591,174-57,603,418
lipid metabolism	Internal & Subcutaneous	BTA5, 56 Mb	*B4GALNT1*	beta-1,4-N-acetyl-galactosaminyltransferase 1	12:57,623,409-57,633,239
lipid metabolism	Internal & Subcutaneous	BTA5, 56 Mb	*CYP27B1*	cytochrome P450 family 27 subfamily B member 1	12:57,762,334-57,768,986
lipid metabolism	IMF & Subcutaneous	BTA6, 36.5 Mb	*PPM1K*	protein phosphatase, Mg2+/Mn2+ dependent 1K	4:88,257,620-88,284,769
lipid metabolism	IMF & Subcutaneous	BTA6, 36.5 Mb	*FAM13A*	family with sequence similarity 13 member A	4:88,725,955-89,111,398
lipid metabolism	IMF	BTA12, 30 Mb	*ALOX5AP*	arachidonate 5-lipoxygenase activating protein	13:30,713,478-30,764,426
lipid metabolism (steroidogenesis)	Internal & Subcutaneous	BTA5, 56.5 Mb	*RDH16*	retinol dehydrogenase 16	12:56,951,431-56,959,374
lipid metabolism (steroidogenesis)	Internal & Subcutaneous	BTA5, 56.5 Mb	*HSD17B6*	hydroxysteroid (17-beta) dehydrogenase 6	12:56,752,161-56,787,790

Supplementary Table S5. Genes in overlapping Meta-QTL for different fat trait groups with candidate genes highlighted.

Ensembl gene id	Gene symbol	Gene name	BTA	Position (Mb)	Meta-QTL ID
ENSBTAG00000042866	*SNORA19*	small nucleolar RNA SNORA19	2	3.0	327
ENSBTAG00000049463	Predicted genes		2	3.5	328
ENSBTAG00000060094	Predicted genes		2	3.5	328
ENSBTAG00000065936	Predicted genes		2	3.5	328
ENSBTAG00000003942	*PIP4K2C*	phosphatidylinositol-5-phosphate 4-kinase type 2 gamma	5	56.0	1196
ENSBTAG00000004494	*B4GALNT1*	beta-1,4-N-acetyl-galactosaminyltransferase 1	5	56.0	1196
ENSBTAG00000006324	*NAB2*	NGFI-A binding protein 2	5	56.0	1196
ENSBTAG00000006335	*STAT6*	signal transducer and activator of transcription 6	5	56.0	1196
ENSBTAG00000006599	*INHBE*	inhibin subunit beta E	5	56.0	1196
ENSBTAG00000006631	*GLI1*	GLI family zinc finger 1	5	56.0	1196
ENSBTAG00000007152	*OS9*	OS9 endoplasmic reticulum lectin	5	56.0	1196
ENSBTAG00000007156	*AGAP2*	ArfGAP with GTPase domain, ankyrin repeat and PH domain 2	5	56.0	1196
ENSBTAG00000007158	*TSPAN31*	tetraspanin 31	5	56.0	1196
ENSBTAG00000007160	*CDK4*	cyclin dependent kinase 4	5	56.0	1196
ENSBTAG00000010616	*MBD6*	methyl-CpG binding domain protein 6	5	56.0	1196
ENSBTAG00000010624	*DCTN2*	dynactin subunit 2	5	56.0	1196
ENSBTAG00000010830	*LRP1*	LDL receptor related protein 1	5	56.0	1196

ENSBTAG00000014655	*MYO1A*	myosin IA	5	56.0	1196
ENSBTAG00000014659	*NEMP1*	nuclear envelope integral membrane protein 1	5	56.0	1196
ENSBTAG00000016508	*CTDSP2*	CTD small phosphatase 2	5	56.0	1196
ENSBTAG00000016904	*MARCHF9*	membrane associated ring-CH-type finger 9	5	56.0	1196
ENSBTAG00000016906	*CYP27B1*	cytochrome P450 family 27 subfamily B member 1	5	56.0	1196
ENSBTAG00000016908	*METTL1*	methyltransferase 1, tRNA methylguanosine	5	56.0	1196
ENSBTAG00000016910	*EEF1AKMT3*	EEF1A lysine methyltransferase 3	5	56.0	1196
ENSBTAG00000016912	*TSFM*	Ts translation elongation factor, mitochondrial	5	56.0	1196
ENSBTAG00000016913	*AVIL*	advillin	5	56.0	1196
ENSBTAG00000017543	*ATP23*	ATP23 metallopeptidase and ATP synthase assembly factor homolog	5	56.0	1196
ENSBTAG00000018002	*DTX3*	deltex E3 ubiquitin ligase 3	5	56.0	1196
ENSBTAG00000018003	*ARHGEF25*	Rho guanine nucleotide exchange factor 25	5	56.0	1196
ENSBTAG00000018358	*STAC3*	SH3 and cysteine rich domain 3	5	56.0	1196
ENSBTAG00000018361	*R3HDM2*	R3H domain containing 2	5	56.0	1196
ENSBTAG00000018403	*ARHGAP9*	Rho GTPase activating protein 9	5	56.0	1196
ENSBTAG00000018405	*MARS1*	methionyl-tRNA synthetase 1	5	56.0	1196
ENSBTAG00000019084	*INHBC*	inhibin subunit beta C	5	56.0	1196
ENSBTAG00000021336	*KIF5A*	kinesin family member 5A	5	56.0	1196
ENSBTAG00000021807	*TAC3*	tachykinin 3	5	56.0	1196
ENSBTAG00000023289	*SLC26A10*	solute carrier family 26 member 10	5	56.0	1196
ENSBTAG00000031500	*SHMT2*	serine hydroxymethyltransferase 2	5	56.0	1196

ENSBTAG00000031503	*NDUFA4L2*	NDUFA4 mitochondrial complex associated like 2	5	56.0	1196
ENSBTAG00000031544	*DDIT3*	DNA damage inducible transcript 3	5	56.0	1196
ENSBTAG00000038498	*ZBTB39*	zinc finger and BTB domain containing 39	5	56.0	1196
ENSBTAG00000039157	*GPR182*	G protein-coupled receptor 182	5	56.0	1196
ENSBTAG00000047650	*NXPH4*	neurexophilin 4	5	56.0	1196
ENSBTAG00000029869	*bta-mir-26a-2*	bta-mir-26a-2	5	56.0	1196
ENSBTAG00000043214	*SNORA62*	Small nucleolar RNA SNORA62/SNORA6 family	5	56.0	1196
ENSBTAG00000044363	bta-mir-2430	bta-mir-2430	5	56.0	1196
ENSBTAG00000045218	bta-mir-2431	bta-mir-2431	5	56.0	1196
ENSBTAG00000051574	Predicted gene		5	56.0	1196
ENSBTAG00000051593	Predicted gene		5	56.0	1196
ENSBTAG00000056492	Predicted gene		5	56.0	1196
ENSBTAG00000057914	Predicted gene		5	56.0	1196
ENSBTAG00000058602	Predicted gene		5	56.0	1196
ENSBTAG00000058760	Predicted gene		5	56.0	1196
ENSBTAG00000059109	Predicted gene		5	56.0	1196
ENSBTAG00000066508	Predicted gene		5	56.0	1196
ENSBTAG00000067979	Predicted gene		5	56.0	1196
ENSBTAG00000043669	*U6*	U6 spliceosomal RNA	5	56.0	1196
ENSBTAG00000031076	Predicted gene		5	56.0	1196
ENSBTAG00000001392	*RDH16*	retinol dehydrogenase 16	5	56.5	1197
ENSBTAG00000004380	*STAT2*	signal transducer and activator of transcription 2	5	56.5	1197
ENSBTAG00000004384	*APOF*	apolipoprotein F	5	56.5	1197
ENSBTAG00000009281	*SPRYD4*	SPRY domain containing 4	5	56.5	1197
ENSBTAG00000009284	*GLS2*	glutaminase 2	5	56.5	1197
ENSBTAG00000010127	*MIP*	major intrinsic protein of lens fiber	5	56.5	1197

ENSBTAG00000010701	*NACA*	nascent polypeptide associated complex subunit alpha	5	56.5	1197
ENSBTAG00000011720	*HSD17B6*	hydroxysteroid (17-beta) dehydrogenase 6	5	56.5	1197
ENSBTAG00000013315	*ATP5F1B*	ATP synthase F1 subunit beta	5	56.5	1197
ENSBTAG00000013443	*SDR9C7*	short chain dehydrogenase/reductase family 9C member 7	5	56.5	1197
ENSBTAG00000017830	*RBMS2*	RNA binding motif single stranded interacting protein 2	5	56.5	1197
ENSBTAG00000017840	*BAZ2A*	bromodomain adjacent to zinc finger domain 2A	5	56.5	1197
ENSBTAG00000017967	*PTGES3*	prostaglandin E synthase 3	5	56.5	1197
ENSBTAG00000020459	*TIMELESS*	timeless circadian regulator	5	56.5	1197
ENSBTAG00000044000	*PRIM1*	DNA primase subunit 1	5	56.5	1197
ENSBTAG00000045969	*APON*	apolipoprotein N	5	56.5	1197
ENSBTAG00000042169	Predicted gene		5	56.5	1197
ENSBTAG00000042677	*bta-mir-677*	bta-mir-677	5	56.5	1197
ENSBTAG00000043048	*SNORD59A*	Small nucleolar RNA SNORD59	5	56.5	1197
ENSBTAG00000043104	Predicted gene		5	56.5	1197
ENSBTAG00000049930	Predicted gene		5	56.5	1197
ENSBTAG00000056353	Predicted gene		5	56.5	1197
ENSBTAG00000062839	Predicted gene		5	56.5	1197
ENSBTAG00000065195	Predicted gene		5	56.5	1197
ENSBTAG00000065825	Predicted gene		5	56.5	1197
ENSBTAG00000051761	Predicted gene		5	56.5	1197
ENSBTAG00000000470	*IBSP*	integrin binding sialoprotein	6	36.5	1400
ENSBTAG00000005260	*SPP1*	secreted phosphoprotein 1	6	36.5	1400
ENSBTAG00000005754	*PPM1K*	protein phosphatase, Mg2+/Mn2+ dependent 1K	6	36.5	1400

ENSBTAG00000007568	*MEPE*	matrix extracellular phosphoglycoprotein	6	36.5	1400
ENSBTAG00000010120	*HERC3*	HECT and RLD domain containing E3 ubiquitin protein ligase 3	6	36.5	1400
ENSBTAG00000010128	*NAP1L5*	nucleosome assembly protein 1 like 5	6	36.5	1400
ENSBTAG00000011187	*FAM13A*	family with sequence similarity 13 member A	6	36.5	1400
ENSBTAG00000017704	*ABCG2*	ATP binding cassette subfamily G member 2	6	36.5	1400
ENSBTAG00000020031	*PKD2*	polycystin 2, transient receptor potential cation channel	6	36.5	1400
ENSBTAG00000020536	*HERC6*	HECT and RLD domain containing E3 ubiquitin protein ligase family member 6	6	36.5	1400
ENSBTAG00000020538	*HERC5*	HECT and RLD domain containing E3 ubiquitin protein ligase 5	6	36.5	1400
ENSBTAG00000020541	*PYURF*	phosphatidylinositol glycan anchor biosynthesis class Y	6	36.5	1400
ENSBTAG00000065096	Predicted gene		6	36.5	1400
ENSBTAG00000065916			6	36.5	1400
ENSBTAG00000005932	*FAM184B*	family with sequence similarity 184 member B	6	37.0	1401
ENSBTAG00000005989	*LAP3*	leucine aminopeptidase 3	6	37.0	1401
ENSBTAG00000019441	*MED28*	mediator complex subunit 28	6	37.0	1401
ENSBTAG00000021582	*NCAPG*	non-SMC condensin I complex subunit G	6	37.0	1401
ENSBTAG00000046561	*LCORL*	ligand dependent nuclear receptor corepressor like	6	37.0	1401
ENSBTAG00000052418	Predicted gene		6	37.0	1401
ENSBTAG00000062387	*DCAF16*	DDB1 and CUL4 associated factor 16	6	37.0	1401

ENSBTAG00000067415	Predicted gene	6	37.0	1401
ENSBTAG00000069386	Predicted gene	6	37.0	1401
ENSBTAG00000057197	Predicted gene	6	37.5	1402

Supplementary Table S6. Genes in Meta-QTL for internal fat on BTA 5.

Ensembl gene id	Gene symbol	Gene name	position (Mb)	Meta-QTL ID
ENSBTAG00000003942	*PIP4K2C*	phosphatidylinositol-5-phosphate 4-kinase type 2 gamma	56.0	1196
ENSBTAG00000004494	*B4GALNT1*	beta-1,4-N-acetyl-galactosaminyltransferase 1	56.0	1196
ENSBTAG00000006324	*NAB2*	NGFI-A binding protein 2	56.0	1196
ENSBTAG00000006335	*STAT6*	signal transducer and activator of transcription 6	56.0	1196
ENSBTAG00000006599	*INHBE*	inhibin subunit beta E	56.0	1196
ENSBTAG00000006631	*GLI1*	GLI family zinc finger 1	56.0	1196
ENSBTAG00000007152	*OS9*	OS9 endoplasmic reticulum lectin	56.0	1196
ENSBTAG00000007156	*AGAP2*	ArfGAP with GTPase domain, ankyrin repeat and PH domain 2	56.0	1196
ENSBTAG00000007158	*TSPAN31*	tetraspanin 31	56.0	1196
ENSBTAG00000007160	*CDK4*	cyclin dependent kinase 4	56.0	1196
ENSBTAG00000010616	*MBD6*	methyl-CpG binding domain protein 6	56.0	1196
ENSBTAG00000010624	*DCTN2*	dynactin subunit 2	56.0	1196
ENSBTAG00000010830	*LRP1*	LDL receptor related protein 1	56.0	1196
ENSBTAG00000014655	*MYO1A*	myosin IA	56.0	1196
ENSBTAG00000014659	*NEMP1*	nuclear envelope integral membrane protein 1	56.0	1196
ENSBTAG00000016904	*MARCHF9*	membrane associated ring-CH-type finger 9	56.0	1196
ENSBTAG00000016906	*CYP27B1*	cytochrome P450 family 27 subfamily B member 1	56.0	1196
ENSBTAG00000016908	*METTL1*	methyltransferase 1, tRNA methylguanosine	56.0	1196
ENSBTAG00000016910	*EEF1AKMT3*	EEF1A lysine methyltransferase 3	56.0	1196
ENSBTAG00000016912	*TSFM*	Ts translation elongation factor, mitochondrial	56.0	1196
ENSBTAG00000016913	*AVIL*	advillin	56.0	1196
ENSBTAG00000017543	*ATP23*	ATP23 metallopeptidase and ATP synthase assembly factor homolog	56.0	1196
ENSBTAG00000018002	*DTX3*	deltex E3 ubiquitin ligase 3	56.0	1196
ENSBTAG00000018003	*ARHGEF25*	Rho guanine nucleotide exchange factor 25	56.0	1196
ENSBTAG00000018358	*STAC3*	SH3 and cysteine rich domain 3	56.0	1196
ENSBTAG00000018361	*R3HDM2*	R3H domain containing 2	56.0	1196
ENSBTAG00000018403	*ARHGAP9*	Rho GTPase activating protein 9	56.0	1196
ENSBTAG00000018405	*MARS1*	methionyl-tRNA synthetase 1	56.0	1196

ENSBTAG00000019084	*INHBC*	inhibin subunit beta C	56.0	1196
ENSBTAG00000021336	*KIF5A*	kinesin family member 5A	56.0	1196
ENSBTAG00000021807	*TAC3*	tachykinin 3	56.0	1196
ENSBTAG00000023289	*SLC26A10*	solute carrier family 26 member 10	56.0	1196
ENSBTAG00000029869	*bta-mir-26a-2*	bta-mir-26a-2	56.0	1196
ENSBTAG00000031076	Predicted gene		56.0	1196
ENSBTAG00000031500	*SHMT2*	serine hydroxymethyltransferase 2	56.0	1196
ENSBTAG00000031503	*NDUFA4L2*	NDUFA4 mitochondrial complex associated like 2	56.0	1196
ENSBTAG00000031544	*DDIT3*	DNA damage inducible transcript 3	56.0	1196
ENSBTAG00000038498	*ZBTB39*	zinc finger and BTB domain containing 39	56.0	1196
ENSBTAG00000039157	*GPR182*	G protein-coupled receptor 182	56.0	1196
ENSBTAG00000043214	*SNORA62*	Small nucleolar RNA SNORA62/SNORA6 family	56.0	1196
ENSBTAG00000044363	bta-mir-2430	bta-mir-2430	56.0	1196
ENSBTAG00000045218	bta-mir-2431	bta-mir-2431	56.0	1196
ENSBTAG00000047650	*NXPH4*	neurexophilin 4	56.0	1196
ENSBTAG00000049045	Predicted gene		56.0	1196
ENSBTAG00000049081	Predicted gene		56.0	1196
ENSBTAG00000050051	Predicted gene		56.0	1196
ENSBTAG00000050652	Predicted gene		56.0	1196
ENSBTAG00000051574	Predicted gene		56.0	1196
ENSBTAG00000051593	Predicted gene		56.0	1196
ENSBTAG00000056492	Predicted gene		56.0	1196
ENSBTAG00000057914	Predicted gene		56.0	1196
ENSBTAG00000058602	Predicted gene		56.0	1196
ENSBTAG00000058760	Predicted gene		56.0	1196
ENSBTAG00000059109	Predicted gene		56.0	1196
ENSBTAG00000061283	*CTDSP2*	CTD small phosphatase 2	56.0	1196
ENSBTAG00000066508	Predicted gene		56.0	1196
ENSBTAG00000067979	Predicted gene		56.0	1196
ENSBTAG00000001392	*RDH16*	retinol dehydrogenase 16 (all-trans)	56.5	1197

ENSBTAG00000004380	*STAT2*	signal transducer and activator of transcription 2	56.5	1197
ENSBTAG00000004384	*APOF*	apolipoprotein F	56.5	1197
ENSBTAG00000009281	*SPRYD4*	SPRY domain containing 4	56.5	1197
ENSBTAG00000009284	*GLS2*	glutaminase 2	56.5	1197
ENSBTAG00000010127	*MIP*	major intrinsic protein of lens fiber	56.5	1197
ENSBTAG00000010701	*NACA*	nascent polypeptide associated complex subunit alpha	56.5	1197
ENSBTAG00000011720	*HSD17B6*	hydroxysteroid (17-beta) dehydrogenase 6	56.5	1197
ENSBTAG00000013315	*ATP5F1B*	ATP synthase F1 subunit beta	56.5	1197
ENSBTAG00000013443	*SDR9C7*	short chain dehydrogenase/reductase family 9C member 7	56.5	1197
ENSBTAG00000017830	*RBMS2*	RNA binding motif single stranded interacting protein 2	56.5	1197
ENSBTAG00000017840	*BAZ2A*	bromodomain adjacent to zinc finger domain 2A	56.5	1197
ENSBTAG00000017967	*PTGES3*	prostaglandin E synthase 3	56.5	1197
ENSBTAG00000020459	*TIMELESS*	timeless circadian regulator	56.5	1197
ENSBTAG00000042169	Predicted gene	Small nucleolar RNA U2-19	56.5	1197
ENSBTAG00000042677	*bta-mir-677*	bta-mir-677	56.5	1197
ENSBTAG00000043048	*SNORD59A*	Small nucleolar RNA SNORD59	56.5	1197
ENSBTAG00000043104	Predicted gene	Small nucleolar RNA U2-30	56.5	1197
ENSBTAG00000044000	*PRIM1*	DNA primase subunit 1	56.5	1197
ENSBTAG00000049930	*bta-mir-677*	bta-mir-677	56.5	1197
ENSBTAG00000051761	Predicted gene		56.5	1197
ENSBTAG00000055984	*APON*	apolipoprotein N	56.5	1197
ENSBTAG00000056353	Predicted gene		56.5	1197
ENSBTAG00000062839	Predicted gene		56.5	1197
ENSBTAG00000065195	Predicted gene		56.5	1197
ENSBTAG00000065825	Predicted gene		56.5	1197

Supplementary Table S7. Genes in Meta-QTL for subcutaneous fat on BTA 6.

Ensembl gene id	Gene symbol	Gene name	position (Mb)	Meta-QTL ID
ENSBTAG00000065668	*MARCHF1*	membrane associated ring-CH-type finger 1	1.0	1329
ENSBTAG00000068079	Predicted gene		1.0	1329
ENSBTAG00000056949	Predicted gene		1.5	1330
ENSBTAG00000000470	*IBSP*	integrin binding sialoprotein	36.5	1400
ENSBTAG00000005260	*SPP1*	secreted phosphoprotein 1	36.5	1400
ENSBTAG00000005754	*PPM1K*	protein phosphatase, Mg2+/Mn2+ dependent 1K	36.5	1400
ENSBTAG00000007568	*MEPE*	matrix extracellular phosphoglycoprotein	36.5	1400
ENSBTAG00000010120	*HERC3*	HECT and RLD domain containing E3 ubiquitin protein ligase 3	36.5	1400
ENSBTAG00000010128	*NAP1L5*	nucleosome assembly protein 1 like 5	36.5	1400
ENSBTAG00000011187	*FAM13A*	family with sequence similarity 13 member A	36.5	1400
ENSBTAG00000017704	*ABCG2*	ATP binding cassette subfamily G member 2	36.5	1400
ENSBTAG00000020031	*PKD2*	polycystin 2, transient receptor potential cation channel	36.5	1400
ENSBTAG00000020536	*HERC6*	HECT and RLD domain containing E3 ubiquitin protein ligase family member 6	36.5	1400
ENSBTAG00000020538	*HERC5*	HECT and RLD domain containing E3 ubiquitin protein ligase 5	36.5	1400
ENSBTAG00000020541	*PYURF*	phosphatidylinositol glycan anchor biosynthesis class Y	36.5	1400
ENSBTAG00000055852	Predicted gene		36.5	1400
ENSBTAG00000060730	Predicted gene		36.5	1400
ENSBTAG00000061490	Predicted gene		36.5	1400
ENSBTAG00000062926	*bta-mir-10170*	bta-mir-10170	36.5	1400
ENSBTAG00000065096	Predicted gene		36.5	1400
ENSBTAG00000065916	Predicted gene		36.5	1400
ENSBTAG00000066212	*U6*	U6 spliceosomal RNA	36.5	1400

ENSBTAG00000005932	*FAM184B*	family with sequence similarity 184 member B	37.0	1401
ENSBTAG00000005989	*LAP3*	leucine aminopeptidase 3	37.0	1401
ENSBTAG00000019441	*MED28*	mediator complex subunit 28	37.0	1401
ENSBTAG00000021582	*NCAPG*	non-SMC condensin I complex subunit G	37.0	1401
ENSBTAG00000046561	*LCORL*	ligand dependent nuclear receptor corepressor like	37.0	1401
ENSBTAG00000052418	Predicted gene		37.0	1401
ENSBTAG00000056786	Predicted gene		37.0	1401
ENSBTAG00000059269	Predicted gene		37.0	1401
ENSBTAG00000062387	*DCAF16*	DDB1 and CUL4 associated factor 16	37.0	1401
ENSBTAG00000067415	Predicted gene		37.0	1401
ENSBTAG00000069386	Predicted gene		37.0	1401
ENSBTAG00000057197	Predicted gene		37.5	1402
